# Comparative analysis of complete plastid genomes from *Lilium lankongense* Franchet and its closely related species and screening of *Lilium*-specific primers

**DOI:** 10.7717/peerj.10964

**Published:** 2021-03-05

**Authors:** Danmei Su, Fumin Xie, Haiying Liu, Dengfeng Xie, Juan Li, Xingjin He, Xianlin Guo, Songdong Zhou

**Affiliations:** Key Laboratory of Bio-Resources and Eco-Environment of Ministry of Education, College of Life Sciences, Sichuan University, Chengdu, Sichuan, China

**Keywords:** *Lilium*, *Lilium*-specific primers, *Lilium lankongense*, Phylogeny, Plastid genome

## Abstract

*Lilium* *lankongense* Franchet is a lily species found on the Qinghai-Tibet Plateau. It is pink with deep red spots, has a high ornamental value, and is used in hybrid breeding of horticultural lily varieties. We have insufficient knowledge of the genetic resources of* L. lankongense* and its phylogenetic relationships with related species. Recent molecular phylogenetic studies have shown a very close phylogenetic relationship between *L. lankongense* and the five species *L. duchartrei*, *L. stewartianum*, *L. matangense*, *L. lophophorum,* and *L. nanum*. However, molecular markers still lack sufficient signals for population-level research of the genus *Lilium*. We sequenced and compared the complete plastid sequences of *L. lankongense* and its five related species. The genomes ranged from 152,307 bp to 152,611 bp. There was a slight inconsistency detected in inverted repeat and single copy boundaries and there were 53 to 63 simple sequence repeats in the six species. Two of the 12 highly variable regions (*t*rnC-*petN* and *rpl32*-*trnL*) were verified in 11 individuals and are promising for population-level studies. We used the complete sequence of 33 plastid genomes, the protein-coding region sequence, and the nuclear ITS sequence to reconstruct the phylogenetic tree of* Lilium* species. Our results showed that the plastid gene tree and nuclear gene tree were not completely congruent, which may be caused by hybridization, insufficient information contained in the nuclear ITS, or the small number of samples. The results of phylogenetic analysis based on plastid genomes indicated that the six *Lilium* species were closely related. Our study provides a preliminarily rebuilt backbone phylogeny that is significant for future molecular and morphological studies of *Lilium*.

## Introduction

Plastids are semi-autonomous organelles that play an extremely important role in photosynthesis. The plastid genome is a circular double-stranded DNA ranging from 120–217 kb (Li et al., 2013; Palmer, 1985). The plastid genomes of most angiosperms exhibit a typical quadripartite structure, composed of large single copy (LSC) and small single copy (SSC) regions, which are separated by two copy regions known as the inverted repeat (IR). The most common structure follows the formulation derived from *Nicotiana tabacum* L. (tobacco). The IR, which is flanked by genes *ycf1* and *trnH*-*GUG,* is designated as IR_A_ and the other IR as IR_B_ (genes *rps*19 and *ndh*F are on either side), the junctions between IR and LSC or SSC are designated as J_LB_(LSC/IR_B_), J_SB_(SSC/IR_B_), J_SA_ (SSC/IR_A_) and J_LA_(LSC/IR_A_) (Shinozaki et al., 1986). The plastid genome is considered to be ideal for studying endangered species conservation (Zhao et al., 2018), relationships of lower taxonomic levels (Kang et al., 2019; Parks, Cronn & Liston, 2009), population genetics studies (Ahmed, 2014) and phylogenetics (Xie et al., 2019b; Xie et al., 2018; Yang et al., 2020) due to its compact size, maternal inheritance, absence of recombination, and low evolutionary rate (Palmer, 1985). The cost of plastid genome sequencing has been reduced by the development of sequencing technologies, providing convenience for related analyses based on the plastid genome. Scholars have developed many molecular markers applicable to population genetics and phylogeny based on the whole plastid genome (Scarcelli et al., 2011; Shaw et al., 2007).

At present, *Lilium* L. (Liliaceae) includes approximately 120 species (Peruzzi, 2016). Almost all of the species are distributed in the temperate and cold regions of the Northern hemisphere and are intermittently distributed in East Asia, Europe, and North America (Liang & Tamura, 2000; MacRae, 1998). Since the development of molecular biology, there have been some discrepancies in the research of classification of the genus *Lilium* based on molecular systematics and morphological characteristics. Most early researchers focused on the classification, origin, evolution, and phylogeny of *Lilium* with the nuclear ITS sequence and proposed suggestions to revise some *Lilium* species (Du et al., 2014; Dubouzet & Shinoda, 1999; Ikinci, Oberprieler & Güner, 2006; Lee et al., 2011; Nishikawa et al., 2001; Nishikawa et al., 1999). Plastid gene fragments have become common molecular markers in the phylogeny within *Lilium*. For example, Hayashi & Kawano (2000) distinguished the evolutionary relationships between *Lilium* and related groups with the *rbcL* and *matK* genes, and determined that the previous division of *Lilium* based on morphological traits should be revised. Due to the limited information contained in plastid gene fragments, researchers usually combine nuclear genes and plastid gene fragments to analyze the subgenus classification and interspecies relationships of *Lilium*. However, the phylogenetic location of some species was unclear if the phylogenetic trees constructed by ITS and plastid genes were incongruous (Gao et al., 2013; Gao, Harris & He, 2015; Givnish et al., 2020; Huang et al., 2018). In the latest research, Givnish et al. (2020) reconstructed phylogenetic trees with 69 *Lilium* whole plastomes and 440 nuclear genes loci of 67 *Lilium* species, and further resolved the phylogenetic relationship of *Lilium*. Although the phylogenetic trees constructed by nuclear genes and plastid genes initially revised the phylogenetic location of some species of *Lilium*, the relationship among related species still needs to be resolved using population-level analyses (Lai et al., 2016; Shen, Zhou & He, 2014). Plastid non-coding regions (intergenic regions and introns) are useful for interspecies and intraspecies analyses due to the different rates of nucleotide substitutions among different taxa. A plastid non-coding region may cause analyses to vary greatly in different taxa (Gielly & Taberlet, 1994; Shaw et al., 2005). It has been found that universal primers are not appropriate for the intraspecific level studies of *Lilium*, especially at the population level (Jiang, 2017; Lai et al., 2016). New *Lilium*-specific primers are needed to amplify suitable plastid fragments for intraspecific phylogeographic and population genetic studies within the genus *Lilium*.

**Figure 1:**
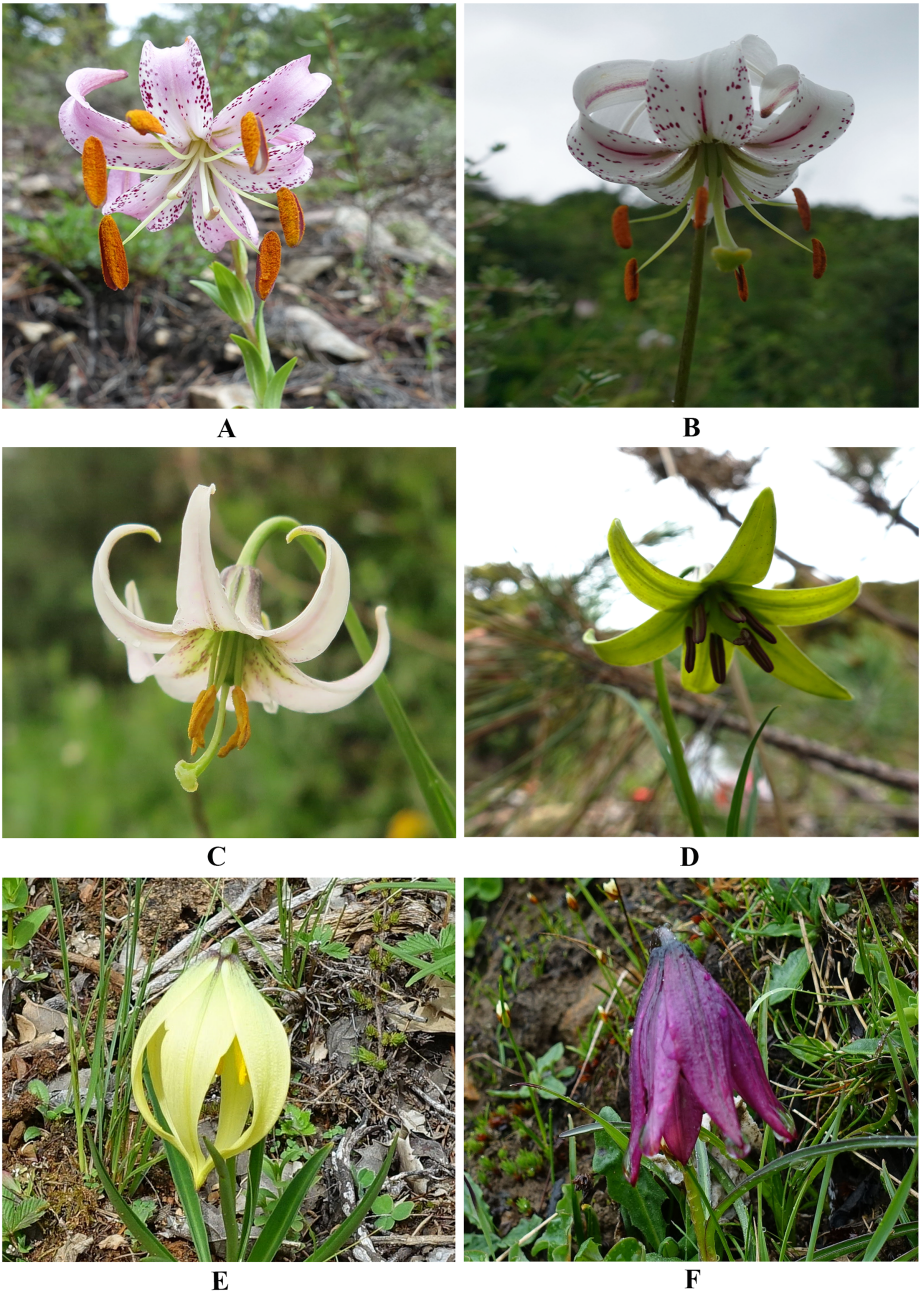
Floral morphology of *L. lankongense* and its five related species. (A) *L. lankongense* (B) *L. duchartrei* (C) *L.* *****matangense* (D) *L.* *****stewartianum* (E)*L.* *****lophophorum* (F) *L. nanum*.

*Lilium lankongense* Franchet has an ovoid-globose bulb, pink tepals with deep red spots, and is endemic to the Qinghai-Tibet Plateau. It has revolute tepals in the margin and nectaries papillose on both surfaces. *L. lankongense* is the parent of hybrids in lily cultivars due to its high resistance to Botrytis blight (an important and very damaging disease of Asiatic hybrids) (Liang & Tamura, 2000; MacRae, 1998; North & Wills, 1969; Proscevičius, Rančeliene & Dambrauskaite, 2007; Van Tuyl et al., 2002). In early molecular and morphological studies, *L. lankongense* was considered to be closely related to *L. duchartrei* Franchet. However, recent molecular evidence has shown that *L. lankongense*, *L. duchartrei*, *L. stewartianum* I.B. Balfour & W.W. Smith, *L. matangense* J.M. Xu, *L. lophophorum* (Bureau & Franchet) Franchet and *L. nanum* Klotzsch, have very close phylogenetic relationships (Gao, Harris & He, 2015; Huang et al., 2018). This is inconsistent with the traditional classification system based on morphological characteristics. *L. lankongense*, *L. duchartrei*, *L. stewartianum*, and *L. matangense* have similar floral features with revolute tepals, while the corolla shape of *L. lophophorum* and *L. nanum* are distinct from the four aforementioned *Lilium* species, which are campanulate (Fig. 1). The other five species possess ovoid to oblong bulbs with multiple scales, and are mainly found in the Qinghai-Tibet Plateau, similar to *L. lankongense* (Gao, Harris & He, 2015; Huang et al., 2018; Liang & Tamura, 2000). Full plastid genomes more accurately reflect interspecies relationships as opposed to single or few plastid DNA (ptDNA) fragments (Yang et al., 2019). Most clades of the plastid genome trees have higher support values in *Lilium* compared with the phylogenetic tree constructed by the nuclear ITS (Liu et al., 2018b). Therefore, it is essential to reconstruct the phylogenetic relationship of *Lilium* based on its plastid genomes. More effective molecular markers also need to be developed to better solve the interspecies relationship of the related species of *Lilium*.

We sequenced, assembled, characterized, and compared the whole plastid genomes of *L. lankongense* and its five related species. We sought to: (1) explore the phylogenetic relationship of *Lilium*, particularly for *L. lankongense*, *L. duchartrei*, *L. stewartianum*, *L. matangense*, *L. lophophorum,* and *L. nanum* using the six plastid genomes and ITS sequences measured in this paper and the published plastid genomes of other *Lilium* species and their corresponding ITS sequences and discuss the possible reasons for the discrepancy between the ITS tree and the plastid gene tree in *Lilium.* (2) Select non-coding regions with relatively more variable loci to develop useful *Lilium*-specific primers for population studies by comparing the six plastid genomes and previously published plastid genomes of other *Lilium* species on the NCBI (https://www.ncbi.nlm.nih.gov/). We provide basic data on plastid genomes for classification, species identification, molecular breeding, biogeography, and genetic diversity in the genus *Lilium*.

## Materials & Methods

### Plant materials and DNA extraction

Leaf samples were collected from each field site (Table S1) and then immediately dried with silica gel to preserve them for DNA extraction. The total genomic DNA was extracted via the NuClean Plant Genomic DNA Kit (ComWin Biosciences, Jiangsu, China) following the manufacturer’s instructions.

### Plastid genome sequencing, assembling, and annotation

All complete plastid genomes were sequenced using the Illumina Novaseq 6000 Platform (Novagene, Beijing, China) with an average paired-end read length of 150 bp. In order to assess the quality of sequenced raw reads, we used the FastQC (Andrews, 2014) v0.11.7. The plastid genome related reads were filtered via mapping after they were assessed for quality and all the raw reads were associated with the previously reported plastid genome sequences in Liliaceae using Bowtie 2 (Langmead & Salzberg, 2012) v2.3.4.3. The published sequence with the highest overall similarity to the reads was used as the seed sequence for further assembly using NOVOPlasty v2.7.2 (Dierckxsens, Mardulyn & Smits, 2017) to obtain a draft sequence. The draft sequence was imported into Geneious v11.0.4 software (Kearse et al., 2012) and was compared with the optimal reference sequence and mapped reads to check and correct for mismatches. The annotation of the plastid genome was also performed in Geneious. Every annotated gene was manually edited for start/stop codons and intron/exon boundaries to correct errors and ensure the accurate annotation of the genome. Finally, circular plastid genome maps were drawn using OGDRAW (Lohse et al., 2013).

### Simple sequence repeats (SSRs) and IR borders analysis

Perl script MISA (Thiel et al., 2003) was used to detect SSRs loci in the six plastid genomes of *Lilium* with thresholds of 10, 5, 4, 3, 3, and 3 for mono-, di-, tri-, tetra-, penta-, and hexanucleotides, respectively. The IR regions were discovered using the plugin Repeat Finder v1.0 in Geneious and were manually checked. The IR plot of the six *Lilium* species plastid genomes was obtained using an IRscope online program to compare the IR borders and neighboring genes of the six *Lilium* species plastid genomes (Amiryousefi, Hyvönen & Poczai, 2018).

### *Lilium*-specific primers

We generated multiple sequence alignments of the six newly assembled sequences and all available plastid genome sequences in *Lilium* (downloaded from NCBI in October 2019) using MAFFT v1.4.0 under the automatic model selection option (Katoh & Standley, 2013). We developed primers specific for the highly mutational regions in *Lilium* using Geneious after alignment (Kearse et al., 2012). The primers were synthesized by Sangon Biotech, China. 12 primers pairs were designed (Table S2) through a series of experiments and two segments of gene spacers were selected with the goal of identifying those primer pairs that would produce the best results. Primer sequences and polymerase chain reaction (PCR) amplification results were summarized in Table S3. PCR of *trnC-petN* was conducted as follows: initial denaturation at 94 °C (3 min) followed by 33 cycles with denaturation at 94 °C (40 s), annealing at 60 °C (50 s), extension at 72 °C (1 min). PCR for *rpl32*-*trnL* was: initial denaturation at 94 °C (3 min), followed by 33 cycles of denaturation at 94 °C (40 s), annealing at 54 °C (45 s) and elongation at 72 °C (1 min). All reactions ended with a final elongation at 72 °C (10 min) followed by a holding step at 4 °C. All PCR products were sequenced in both directions by Sanger sequencing (Sanger & Coulson, 1975) (performed at Sangon Biotech, China), and then assembled and edited using Seqman v7.1.0 software (DNAstar package; DNAStar Inc., Madison, WI, USA) (Burland, 2000) for obtaining consensus sequences. PCR products of these intergenic spacer regions were obtained for 11 additional accessions belonging to three *Lilium* species, including those from geographically remote populations. *L. lankongense* was represented by five populations (from Sichuan, Yunnan, and Tibet), *L*. *duchartrei* was represented by four populations (from Sichuan), and *L*. *lophophorum* was represented by two populations (from Sichuan and Yunnan); each of these species had one individual per population.

**Table 1:** Summary of six complete plastid genomes of *Lilium*

Taxon	Full		LSC length (bp)	SSC length (bp)	IR length (bp)	Gene number	Protein-coding	tRNAs	rRNAs	GenBank accesion number
	Length (bp)	GC (%)								
*L. lankongense*	152, 611	37	81, 995	17, 506	26, 555	132	86	38	8	MK757466
*L. duchartrei*	152, 566	37	81, 870	17, 542	26, 577	132	86	38	8	MN745200
*L. stewartianum*	152, 307	37	81, 921	17, 532	26, 427	132	86	38	8	MN745202
*L. matangense*	152, 402	37	82, 107	17, 531	26, 427	132	86	38	8	MN745201
*L. lophophorum*	152, 382	37	82, 150	17, 382	26, 425	132	86	38	8	MK493298
L. *nanum*	152, 417	37	82, 056	17, 505	26, 428	132	86	38	8	MK493300

### Phylogenetic analysis

We combined the 27 published plastid genome sequences (downloaded from NCBI in October 2019) and ITS sequences from the NCBI and the six plastid genome sequences and corresponding ITS sequences measured by this study (GenBank accession number: MT260888 –MT260893, leaf samples shown in Table S1). Two sequences in *Fritillaria* were treated as the outgroups. We adopted the maximum likelihood (ML) and Bayesian inference (BI) methods to analyze the phylogenetic relationship of *Lilium*. The universal primers ITS4 and ITS5 (White et al., 1990) were used to amplify the ITS according to the standard PCR protocols of Gao et al. (2013). A total of three data sets, namely 33 complete plastid genome sequences, all shared protein-coding genes (CDS) (only containing one IR). The 33 plastid genome sequences and ITS sequences of the species corresponding to these 33 plastid genome sequences were used to reconstruct the phylogenetic tree of *Lilium*. We extracted the shared CDS from 33 plastid genome sequences as follows: all CDS were extracted from all 33 sequences in the software Geneious v11.0.4, and shared 71 CDS (excluding additional copies in the IR) of the 33 species were selected and manually sorted. The CDS belonging to one species were concatenated to generate 33 sequences for phylogenetic analysis. The three data sets were aligned by MAFFT v1.4.0 under the automatic model selection option, trimmed via trimAl v1.2 (Capella-Gutiérrez, Silla-Martínez & Gabaldón, 2009) with parameters Trimal -in *.fas -noallgaps -fasta -out *.fas, and were compiled into three alignment matrices. The three alignment matrices were used to search for the best-fit substitution model using ModelFinder plugin in PhyloSuite v1.2.1 software before ML and BI analyses were conducted (Zhang et al., 2020). ML analysis based on the SYM+G (ITS) and GTR + I + G (plastid genome sequences and shared concatenated 71 CDS sequences) model was conducted using RAxML v8.2.8 (Stamatakis, 2014) with 1000 bootstrap replicates. BI analysis based on the GTR + I + G model was conducted using the Mrbayes v3.2.6 plugin in PhyloSuite v1.2.1 software. The Monte Carlo Markov chains (MCMCs) were run 1 × 10^8^ generations and the first 30% of trees were discarded as burn-in. ML tree used Bootstrap support (BS) and BI tree used posterior probability (PP) to evaluate the feasibility of each branch.

## Results

### Plastid features of *Lilium* species

**Figure 2:**
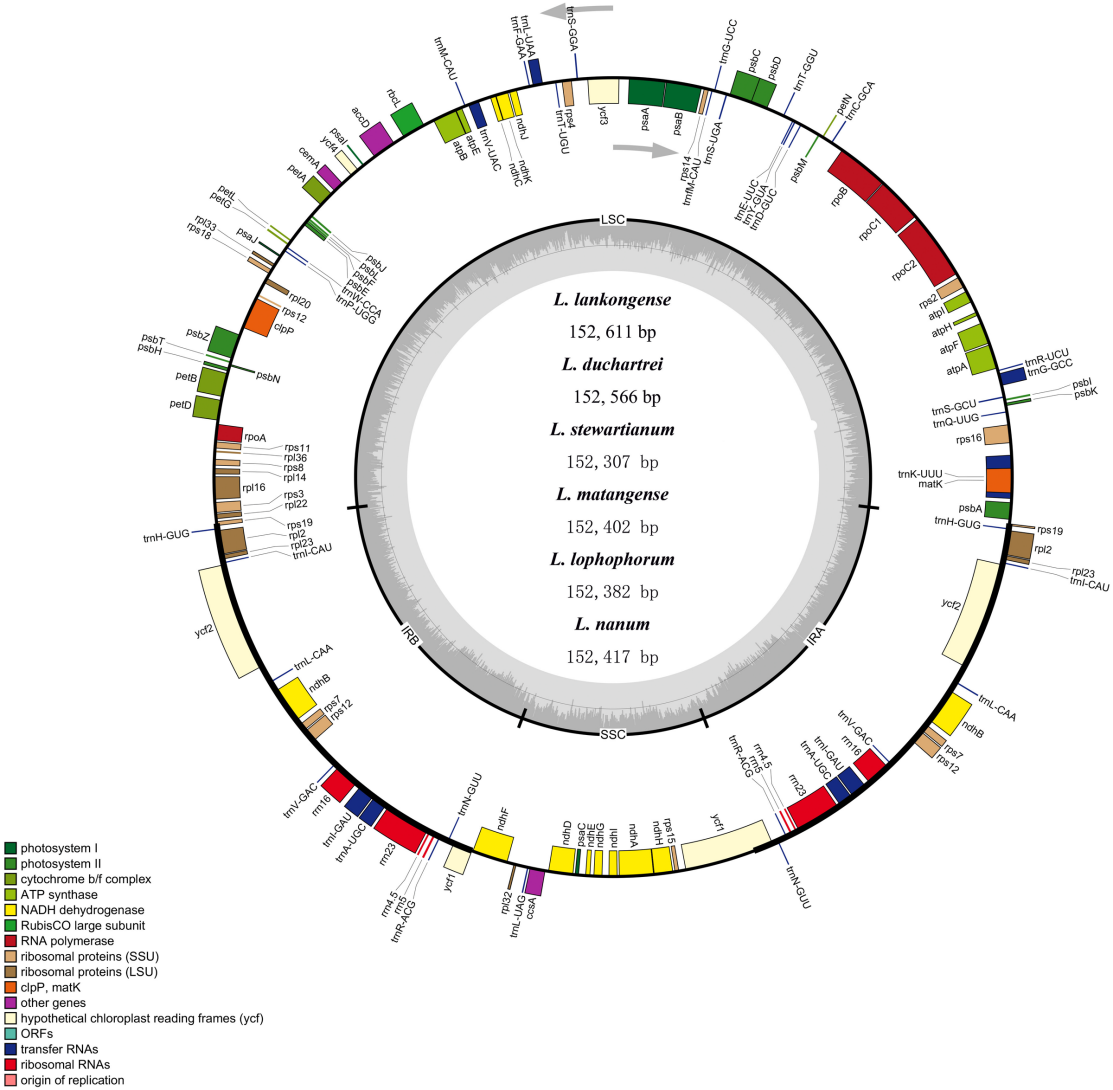
Merged gene map of the complete plastid genomes of six *Lilium* species. Genes belonging to different functional groups are denoted by different colors. The dashed area in the inner circle corresponds to the GC content of the chloroplast genome.

The complete plastid genome of the six species in *Lilium* was deposited in GenBank. These plastid genomes ranged from 152,307 bp (*L. stewartianum*) to 152,611 bp (*L*. *lankongense*) in length, with the minimum and maximum differences being 15 and 304 bp, respectively (Table 1 and Fig. 2). All six plastid genomes displayed a typical quadripartite structure, consisting of the LSC (81, 870–82, 150 bp) and SSC (17, 382–17, 542 bp) regions separated by a pair of IRs (26, 425–26, 577 bp). In the six *Lilium* plastid genomes, the overall GC content was 37%. Both contained 132 genes, with 86 protein-coding genes, 38 tRNA genes, eight ribosomal RNA genes (Table 1 and Table S3).

### Simple sequence repeats (SSRs) analysis

**Figure 3:**
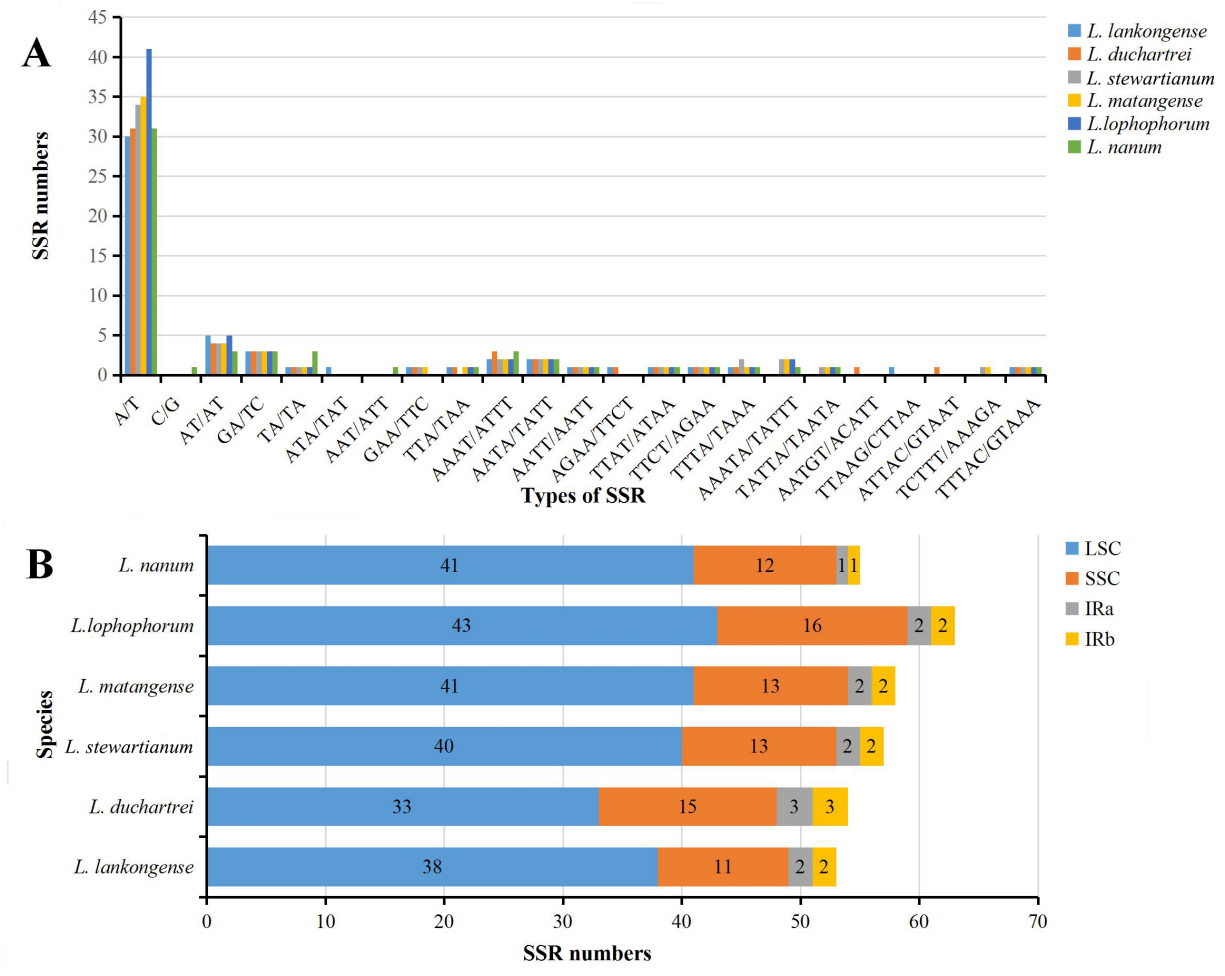
Analysis of simple sequence repeats (SSRs) in six *Lilium* plastid genomes. (A) Number of SSRs in different repeat types; (B) frequency of identified SSRs in different regions.

The SSRs loci in the six plastid genome sequences of *Lilium* were detected using MISA perl script. We detected a total of 340 SSRs in the six *Lilium* plastid genomes, with different numbers of SSRs per species (Table 2). 53, 54, 57, 58, 63, and 55 SSRs were observed in *Lilium lankongense*, *L. duchartrei*, *L. stewartianum*, *L. matangense*, *L. lophophorum*, and *L. nanum* (Table 2, Table S4). *L. lankongense* (53 SSRs) and *L. lophophorum* (63 SSRs) had the lowest and highest number of SSRs, respectively. All SSRs were categorized as mononucleotide, dinucleotide, trinucleotide, tetranucleotide, pentanucleotide, and hexanucleotide (Table 2). The total number of mononucleotide repeats consisting of A or T bases was the largest compared to other types of microsatellites (Fig. 3A) and was greater than the sum of the other types. There were no hexanucleotide repeats found in the six *Lilium* species. In the total SSRs loci, most of the repeats were located in the LSC region, followed by the SSC region and IR regions (Fig. 3B). Additionally, 20 common SSRs were found among these six plastid genome sequences (Table S4), and the number and location of these SSRs were different.

**Table 2:** Types and numbers of SSRs in plastid genomes of the six *Lilium* species.

**Species**	**mononucleotide**	**dinucleotide**	**trinucleotide**	**tetranucleotide**	**pentanucleotide**	**hexanucleotide**	**Total**
*L. lankongense*	30	9	3	9	2	0	53
*L. duchartrei*	31	8	2	10	3	0	54
*L. stewartianum*	34	8	1	9	5	0	57
*L. matangense*	35	8	2	8	5	0	58
*L. lophophorum*	41	9	1	8	4	0	63
*L. nanum*	32	9	2	9	3	0	55
	203	51	11	53	22	0	340

### IR expansion and contraction

**Figure 4:**
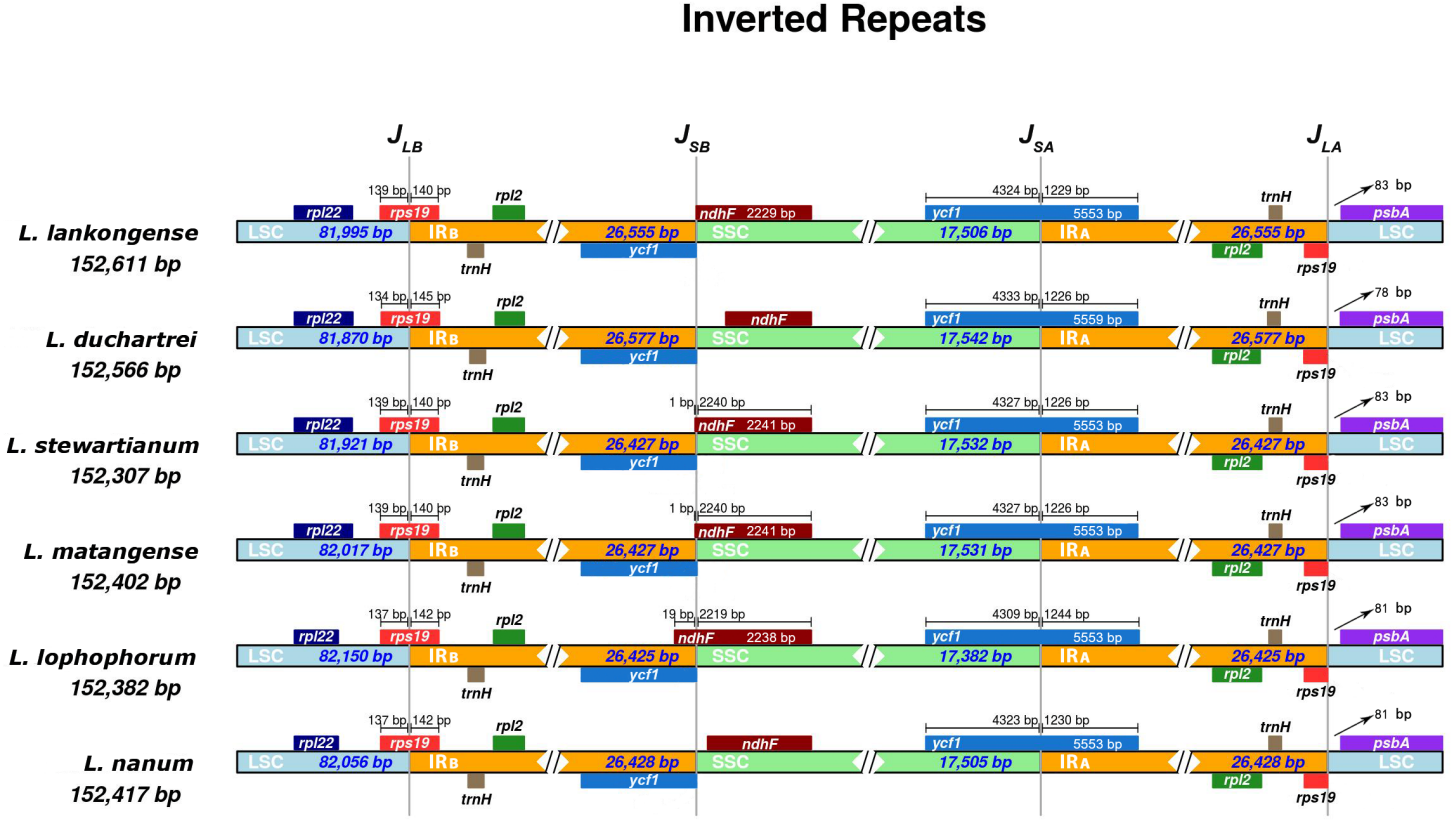
Comparison of the junction positions of large single-copy (LSC), small single-copy (SSC), and inverted repeat (IR) regions among six *Lilium* plastid genomes. Colored boxes for genes indicate the gene position.

The IR boundaries of the six *Lilium* species were shown in Fig. 4. The *rps*19, *ndh*F, *ycf*1, and *psb*A genes were distributed in the J_LB_, J_SB_, J_SA_, and J_LA_ regions, respectively (Fig. 4). The order and types of genes were very conservative. Despite the similar lengths of these six species IR regions (ranging from 26,425 to 26, 577 bp) there is a slight difference in the IR expansion and contraction. The *rps*19 gene crosses the LSC and IR_B_ regions (J_LB_), with 140–145 bp extension into the IR_B_ region. The J_SA_ line intersects the ycf1 gene and the SSC and IR_A_ regions are the same in *Lilium stewartianum* and *L. matangense* (4,327 bp in SSC and 1,226 bp in IR_B_) but are different in other species (4,323 to 4,333 bp in SSC and 1,226 to 1,244 bp in IR_B_) (Fig. 4). The *psb*A gene in the J_LA_ regions remained completely within the LSC region, 78 to 83 bp away from J_LA_ line. The changes in the boundary transformations between the LSC/IR and J_SA_ regions were relatively stable, whereas the J_SB_ regions varied among the six species to some extent. The J_SB_ line is located between *ycf*1 and *ndh*F. Compared to the six *Lilium* species, the J_SB_ region showed that *ndh* F expanded in three species (*L. stewartianum*, *L*. *matangense*, *L*. *lophophorum*), but contracted in two species (*L*. *duchartrei*, *L*. *nanum*) (Fig. 4).

### Development of *Lilium*-specific primers

**Figure 5:**
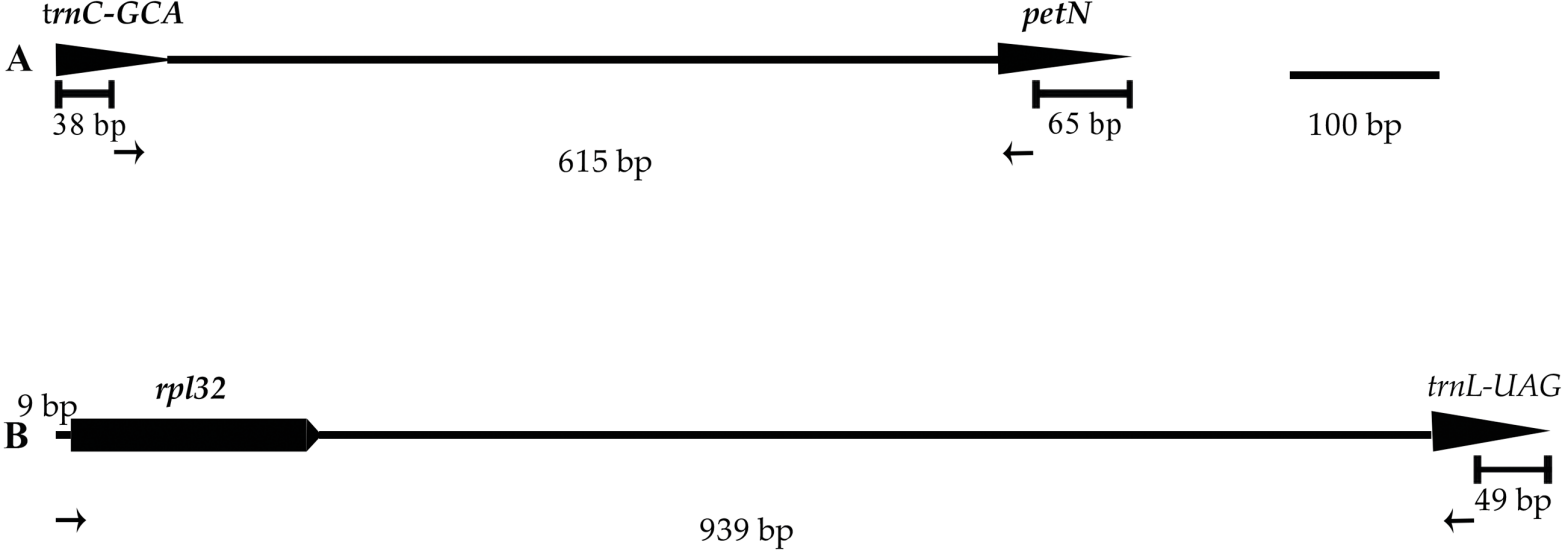
Positions and directions of two new primers used to amplify. Tips of arrows indicate the 3′ ends of the primers. The length of the amplified regions in the figure corresponds to the aligned length of all published chloroplast genome sequences in *Lilium* using MAFFT. (A) *trnC-petN*; (B) *rpl32-trnL*.

Two of the 12 primer pairs designed to sequence the plastid genome of the genus *Lilium* and tested on three species of *Lilium*, amplified consistently and easily produced good quality sequences. The two primer pairs, listed in Table 3 and Fig. 5, were tested on 11 individuals belonging to *Lilium lankongense*, *L*. *duchartrei*, *L*. *lophophorum*, including those from geographically remote populations (Table S5). Comparisons of intergenic spacer regions among *L*. *lankongense*, *L*. *duchartrei*, *L*. *lophophorum* plastomes provided evidence of high variability within *trnC*-*petN*, *rpl32*-*trnL* regions. The 620 bp *trnC*-*petN* and 793 bp *rpl32*-*trnL* regions were aligned for 11 individuals (Table S6, Supplementary Nucleotide alignment *trnC-petN* and Nucleotide alignment *rpl32-trnL*). We found nine, four, and one mutation sites in the *trnC*-*petN* region in *L*. *lankongense*, *L*. *duchartrei*, and *L*. *lophophorum*, respectively. Sequences in the *rpl32*-*trnL* region aligned data set were observed in the different number of variable sites in *L*. *lankongense* (10), *L*. *duchartrei* (4), *L*. *lophophorum* (1) (Table 4 and Table S6).

**Table 3:** Sequences of primers used for PCR amplification and sequencing.

**Region**	**Primer name**	**Sequence (5′-3′)**	**GC (%)**
*trn*C-*pe*tN	*trn*C	CCTTTATCCCCAGTTCAAATCTG	43.48
	*pet*N	CCCAAGCGAGACKTACTATATCCAT	46.00
*rpl*32-*trn*L	*rpl*32	TGTTTTTGAAWGGCGGTTCC	45.00
	*trn*L	CAGCGTGTCTACCAATTTCAC	47.62

**Table 4:** Numbers of variable sites and Genbank Accession Number for the amplified sequences in this study.

**Species**	**Numbers of variable sites**		**Genbank Accession Number**	
	***trnC-petN***	***rpl32-trnL***	***trnC-petN***	***rpl32-trnL***
*L. lophophorum*	1	1	MN764136	MN764115
			MN764135	MN764120
*L. duchartrei*	4	4	MN764128	MN764122
			MN764124	MN764112
			MN764127	MN764113
			MN953785	MN953787
*L. lankongense*	9	10	MN953784	MN953786
			MN764132	MN764116
			MN764134	MN764111
			MN764125	MN764123
			MN764131	MN764114
	13	15		

**Figure 6:**
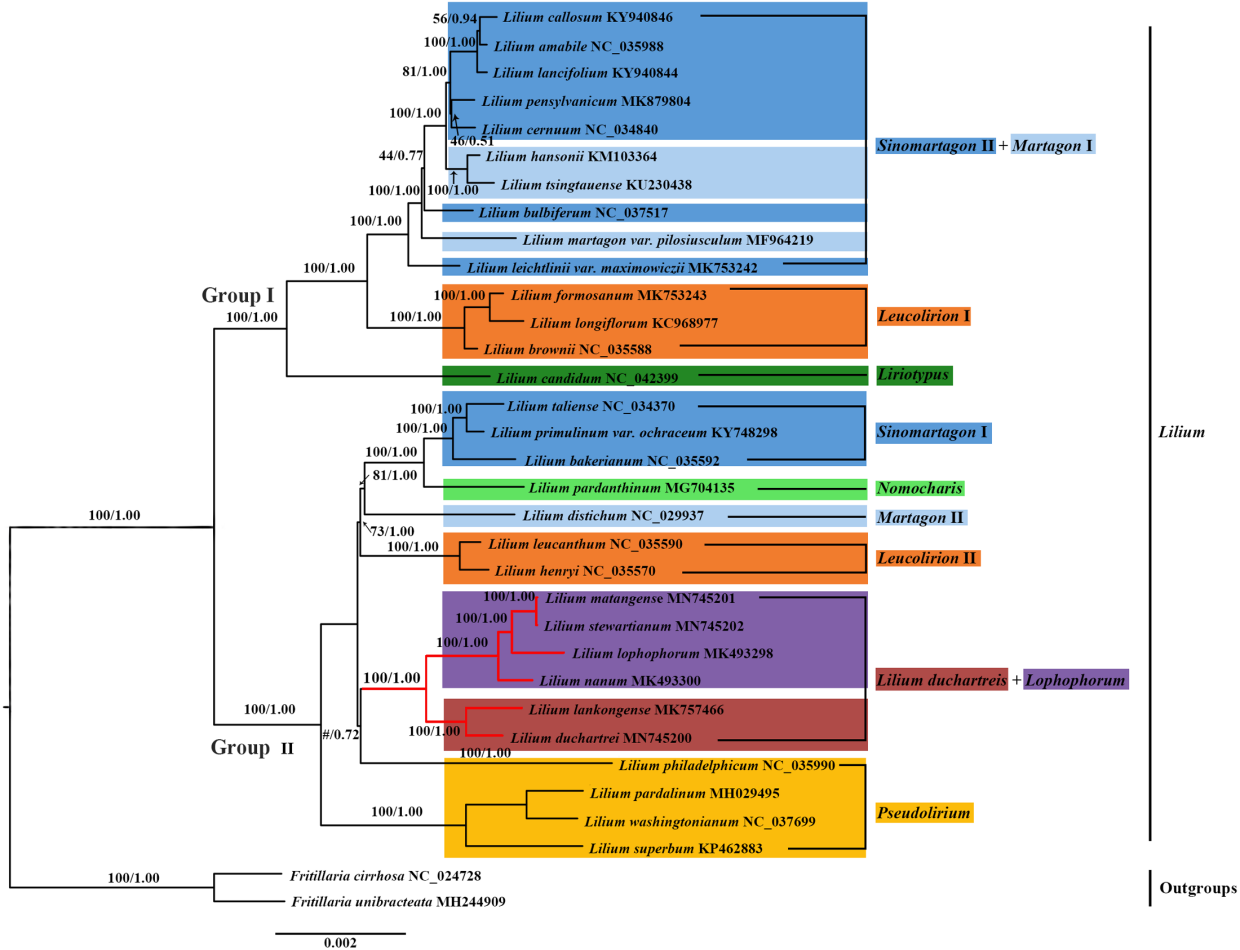
Phylogenetic tree reconstruction including 33 species using Bayesian inference (BI) method based on the plastid genomes. Species of the *L. lankongense* and five related species are highlighted in red branches. The color patches highlight species in each of the traditional sections and Nomoc*haris* or *Lilium duchartreis* clade. Bootstrap support values in the maximum likelihood (ML) trees and posterior probabilities in the BI trees are shown at the corresponding nodes. Nodes that do not occur in the ML tree are indicated by #.

**Figure 7:**
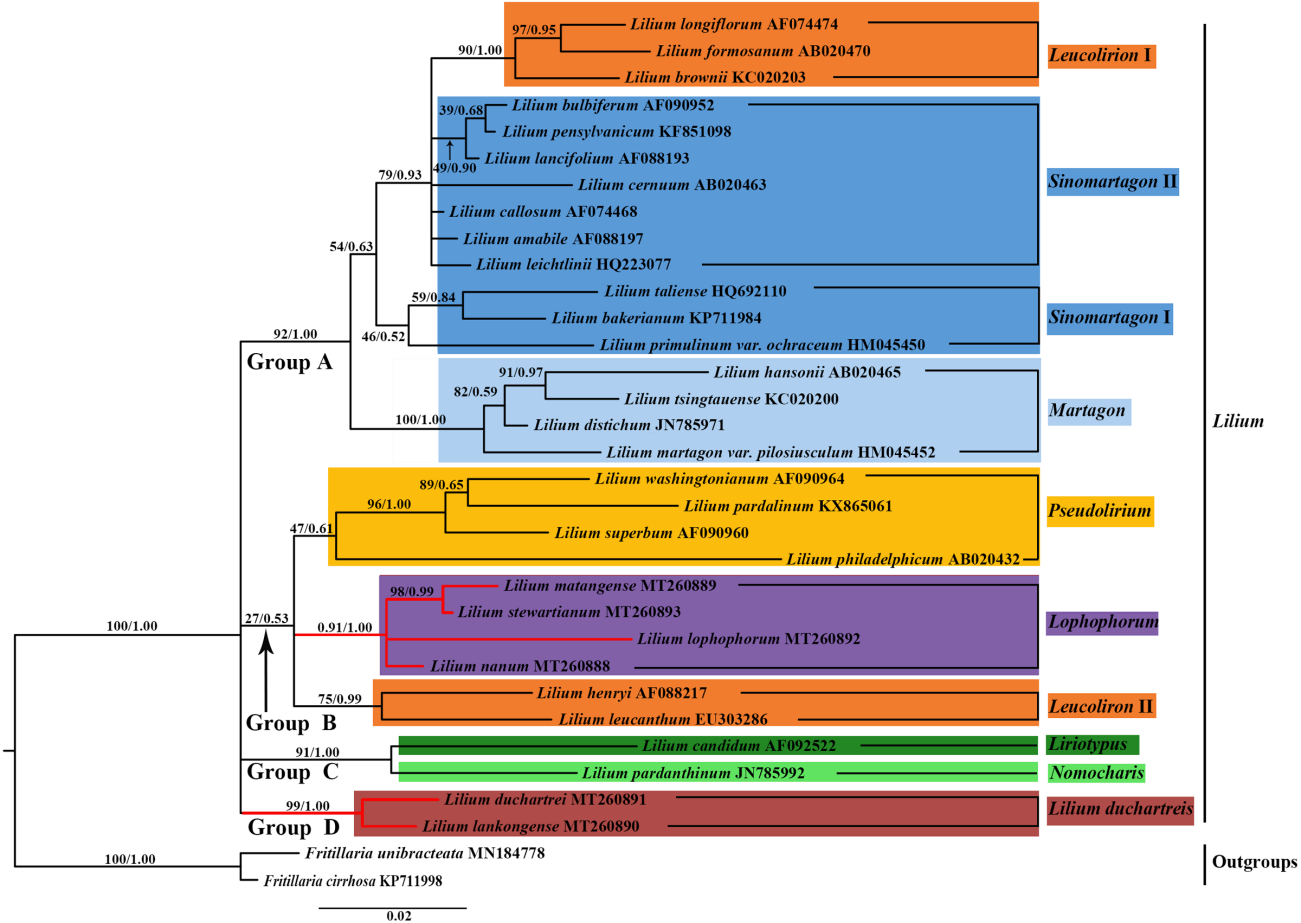
Phylogenetic tree reconstruction including 33 species using Bayesian inference (BI) method based on nuclear ITS. Species of the *L. lankongense* and five related species are highlighted in red branches. The color patches highlight species in each of the traditional sections and Nomoc*haris* or *Lilium duchartreis* clade. Bootstrap support values in the the maximum likelihood (ML) trees and posterior probabilities in the BI trees are shown at the corresponding nodes.

### Phylogenetic analysis

We constructed the phylogenetic tree (containing 31 *Lilium* species, two *Fritillaria* as the outgroups) from the three data sets of the complete plastid genome sequences, the shared 71 CDS sequences, and ITS sequences using the maximum likelihood and Bayesian inference methods. The results are shown in Figs. 6 and 7, and Fig. S1. The phylogenetic trees obtained by ML and the BI methods had identical topologies. The topologies of the phylogenetic tree constructed by ML and BI methods were largely the same based on the complete plastid genome sequences and the shared 71 CDS sequences (Fig. 6 and Fig. S1). The trees strongly supported that *Lilium* was a monophyletic group. The support values of phylogenetic trees constructed by the whole plastid genome sequences were slightly improved when compared with the phylogenetic trees constructed by shared CDS sequences. The phylogenetic relationships of most species were relatively clear and had high support values. Two groups were identified among the 31 *Lilium* species. We considered traditional sections (Comber, 1949; Wang & Tang, 1980) and used *Nomocharis* or *Lilium duchartreis* (including *L. duchartrei and L. lankongense*) as a clade, respectively, for clarification. Group I was composed of 14 species and was divided into the *Sinomartagon* II *+ Martagon* I, *Leucolirion* I, and *Liriotypus* clades. Group II was made up of the remaining 17 species, belonging to the *Lilium duchartreis* + *Lophophorum*, *Sinomartagon* I, *Nomocharis*, *Martagon* II, *Leucolirion* II, and *Pseudolirium* clades. The ML tree and the BI tree also indicated that Sect. *Sinomartagon*, Sect. *Martagon*, Sect. *Leucolirion* and Sect. *Pseudolirium* did not form monophyletic groups. Sect. *Sinomartagon*, Sect. *Martagon* and Sect. *Leucolirion* each formed two clades. *L*. *lankongense*, *L*. *duchartrei*, *L*. *stewartianum*, *L*. *matangense*, *L*. *lophophorum,* and *L*. *nanum* were clustered into the *Lilium duchartreis* + *Lophophorum* branch with strong support (BS = 100%, PP = 1.00). The phylogenetic trees (ML tree and BI tree) reconstructed by ITS sequencing also strongly suggested that *Lilium* was a monophyletic group (Fig. 7). The main structure of *Lilium* was divided into four groups, Group A included the *Leucolirion* I, *Sinomartagon* II, *Sinomartagon* I, and *Martagon* clades, Group B contained the the *Lophophorum*, *Pseudolirium*, and *Leucolirion* II clades, *Nomocharis* and *Liriotypus* clades made up Group C, and the *Lilium duchartreis* clade alone formed Group D. The phylogenetic tree, established by the 33 plastid genomes or the shared 71 CDS, was not completely consistent with the phylogenetic tree constructed by ITS. In the ITS phylogenetic tree, four species in Sect. *Martagon* and four species in Sect. *Pseudolirium* were respectively monophyletic clades. *L*. *lankongense* and *L*. *duchartrei* belonged to *Lilium duchartreis* clade, while *L*. *stewartianum*, *L*. *matangense*, *L*. *lophophorum*, and *L*. *nanum* belonged to another clade (*Lophophorum* clade), and the *Lilium duchartreis* clade and the *Lophophorum* clade were in two different groups. Compared with the phylogenetic trees formed by complete plastid genome sequences and its shared 71 CDS sequences, the ITS phylogenetic tree was more in line with the previous classification based on morphology.

## Discussion

### Comparative analysis of *Lilium* plastid genomes

The plastid genomes of the six *Lilium* species were compared and analyzed. Similar to other *Lilium* species (Du et al., 2017; Kim & Kim, 2013; Liu et al., 2018a; Liu et al., 2018b), the plastid genomes of these six species all have LSC and SSC regions, and a pair of IRS (Table 1). Moreover, these six plastid genomes have the same protein-coding genes, tRNAs, and rRNAs (Table 1), and have the typical structure of land plant plastid genomes (Wicke et al., 2011). Their lengths varied from 152,307 to 152,611 bp (Table 1), indicating a slight variation. The LSC and IR boundaries and the J_SA_ region of the six plastid genomes were relatively stable with small differences, while the J_SB_ region was relatively obvious. The *ndh* F gene of the J_SB_ region showed signs of contraction and expansion. Typically, the IR (IR_A_ and IR_B_) length differs among plant species. In angiosperms, the size variations in the plastid genome are attributed to the expansion and contraction of the IR region and single-copy (SC) border regions (Chumley et al., 2006) and play a vital role in its evolution (Wang et al., 2008). According to our results, the IR expansion and contraction may act as a primary mechanism for the variation in the length of the six plastid genomes. Similar results have been shown in most land plants (Gao et al., 2019; Hansen et al., 2007; Huang et al., 2014).

Microsatellites (SSRs) also known as short tandem repeats (STRs), are usually composed of one to six nucleotide repeat units (Chen et al., 2006; Powell, Machray & Provan, 1996), and are found in the majority of eukaryotic genomes (Tautz & Renz, 1984). SSRs are suitable molecular markers because of their high rate of polymorphism, and they are mostly co-dominant, and highly transferable (Scott et al., 2000). The SSRs in the plastid genome, which were viewed as complements to the nuclear microsatellite markers, were useful in research of phylogeography, population genetics, systematics, and breeding (Gaudeul et al., 2011; Hashimoto et al., 2004; Sánchez-Pérez et al., 2005; Xue, Wang & Zhou, 2012). The repeat units in plastid SSRs of these *Lilium* genomes were mostly comprised of adenine (A) or thymine (T) repeats, but were rarely guanine (G) or cytosine (C) repeats (Fig. 3A), which resulted from the AT-richness of the plastid genome (Zhang et al., 2016). These results are identical to other *Lilium* species (Du et al., 2017; Liu et al., 2018b). We identified a total of 340 SSR information sites. These SSR information sites can provide a reference for the subsequent development and application of SSRs molecular markers within *Lilium*.

### *Lilium*-specific primers

Noncoding regions of the plastid genome play an important molecular role in systematic, phylogeographic, and population genetic studies of angiosperms (Perret et al., 2003; Xie et al., 2019a; Yang et al., 2016). Previous researchers, including Lai et al. (2016) tried to amplify the *trnV*-*ndhC* and *petL*-*petE* regions of the plastid genome but failed to amplify the corresponding fragments of all individuals. Jiang (2017) was unable to find suitable ptDNA fragments by screening 33 published universal primers for their phylogeographic study. No suitable ptDNA fragments from the genus *Lilium* have been found for use in population studies to date. In this study, 12 primers were designed according to the conserved regions of published *Lilium* plastid genomes to amplify a wide range of *Lilium* species. Two of the 12 pairs of primers (*trnC*-*petN* and *rpl32*-*trnL*) could amplify the products in all experimental individuals. The *trnC*-*petN* and *rpl32*-*trnL* regions proved to be effective in phylogeny and phylogeography in other genera (Liu, 2017; Lo Presti & Oberprieler, 2011; Yu et al., 2011) and the two non-coding regions of *trnC*-*petN* and *rpl32*-*trnL* may be used for intraspecies research of *Lilium*. The other 10 primer pairs (Table S2) successfully amplified some individuals, such as *trnQ*-*rps16*, *trnF*-*ndhJ,* and *trnE*-*trnT*, which had similar proportions of variant sites in amplified sequences and were applied to population-level studies in other genera (Chen et al., 2013; Dane & Liu, 2007; Qiu et al., 2009). We suggest that *trnC*-*petN* and *rpl32*-*trnL* with the two new primer pairs can be used in subsequent population studies (Table 3). Future studies should focus on discovering better primers for other potential regions to amplify their products. The two pairs of primers (*trnC*-*petN* and *rpl32*-*trnL*) were selected based on their plastid genomes and provide basic data for the genetic diversity and molecular breeding of this important wild flower of *Lilium*.

### Phylogenetic analysis

We constructed a phylogenetic tree based on 3 data sets (the plastid genome sequences, the shared 71 CDS sequences, and ITS sequences). The tree supported *Lilium* as a monophyletic group with subgroups that include Sect. *Sinomartagon* and Sect. *Leucolirion,* which were both polyphyletic (Figs. 6, 7, and Fig. S1). Sect. *Martagon* and Sect. *Pseudolirium* were both monophyletic in the ITS tree, but none of them formed a monophyletic group in the plastid genome and the shared 71 CDS trees (Figs. 6, 7, and Fig. S1). Givnish et al. (2020) also sequenced the plastomes and 440 nuclear loci of 67 species of *Lilium*, and the results are similar to ours. Previous studies have also shown a conflict between the nuclear gene tree and the plastid gene tree in *Lilium*. This discordance may be due to hybridization in *Lilium* (Gao et al., 2013; Gao, Harris & He, 2015; Givnish et al., 2020; Gong et al., 2017; Huang et al., 2018). Due to the limited dispersion of seeds and the pollination by wind or pollinators, hybridization in *Lilium* is likely to occur among neighboring species. Hybridization may play an important role in *Lilium* flower morphology (Gao, Harris & He, 2015; Givnish et al., 2020). Furthermore, there are paralogous homologs in the ITS sequences of Liliaceae that may not reflect the true interspecific relationships in the phylogenetic tree (Day et al., 2014; Gao et al., 2012b; Huang et al., 2018). The 31 species of *Lilium* studied could not exclude the paralogous homology of the ITS sequences. Finally, *L*. *lankongense*, *L*. *duchartrei*, *L*. *stewartianum*, *L*. *matangense*, *L*. *lophophorum,* and *L*. *nanum* in the ITS tree were split into two different clades. The phylogenetic trees constructed by Huang et al. (2018) based on the ITS of 140 accessions in Liliaceae showed that the six species occur together in a clade. Aside from those results, the phylogenetic trees constructed with 440 nuclear genes and their spacers by Givnish et al. (2020) supported five species (except *L. matangense*), including *L*. *lankongense*, *L*. *duchartrei*, *L*. *stewartianum*, *L*. *lophophorum*, and *L*. *nanum* as a monophyletic group. The nuclear gene phylogenetic tree in this study only involved 33 ITS sequences (the number of samples was small), the information reflected by the ITS sequences was limited, and some clades (such as the *Pseudolirium* and *Sinomartagon* I clades) had low support values. The aforementioned three points are also one of the reasons for discordance between the ITS tree and the plastid gene tree.

In sections delimited by Comber (1949), which were based on morphology, *L*. *lankongense*, *L*. *duchartrei*, *L*. *stewartianum*, *L*. *lophophorum,* and *L*. *nanum* belong to Sect. Sinomartagon. *L*. *lankongense*, *L*. *duchartrei* belong to Sect. *Sinomartagon* 5a, while *L*. *stewartianum*, *L*. *lophophorum*, and *L*. *nanum* belong to Sect. *Sinomartagon* 5c (Comber, 1949). Sect. *Sinomartagon* Comber, which is the largest group of the genus *Lilium*, includes rather disparate species, and various researchers had different treatments for the division of its subsection (Comber, 1949; Du et al., 2014; Gao, Harris & He, 2015; Givnish et al., 2020; Nishikawa et al., 2001). Sect. *Sinomartagon* Comber was divided into the *Sinomartagon*, *Lophophorum*, and *Lilium duchartreis* clades based on the ITS tree (Fig. 7). The species in the *Lophophorum* clade contained *L*. *stewartianum*, *L*. *matangense*, *L*. *lophophorum,* and *L*. *nanum*; the *Lilium duchartreis* clade was composed of *L*. *lankongense* and *L*. *duchartrei*. The plastid gene trees (the plastid genome sequences, the shared 71CDS sequences) and the ITS tree both supported that Sect. *Sinomartagon* Comber was not monophyletic (Figs. 6, 7, and Fig. S1). In the plastid and ITS trees, *L. lankongense* was sister to *L*. *duchartrei*, *L. stewartianum* was evolutionarily close to *L. matangense*. Different phylogenetic relationships were shown among *L*. *lankongense*, *L*. *duchartrei*, *L*. *stewartianum*, *L*. *matangense*, *L*. *lophophorum,* and *L*. *nanum*. These six species were a monophyletic group in the plastid gene trees, and two clades were formed in the ITS tree. Previous studies have shown that *L*. *lankongense* and *L*. *duchartrei* was a monophyletic group, while *L*. *stewartianum*, *L*. *matangense*, *L*. *lophophorum,* and *L*. *nanum* were clustered into another clade in the ITS tree (Gao et al., 2013; Gao, Harris & He, 2015). Huang et al. (2018) constructed a phylogenetic tree based on the ITS sequence, three ptDNA fragments, and the combined (ITS + ptDNA) data sets. All trees supported that the six species were a monophyletic group (Sect. Sinomartagon I). Phylogenetic trees constructed with 440 nuclear genes and their spacers by Givnish et al. (2020) showed that the five species (*L*. *lankongense*, *L*. *duchartrei*, *L*. *stewartianum*, *L*. *lophophorum,* and *L*. *nanum*) aggregated into a single clade. Hence, *L*. *lankongense*, *L*. *duchartrei*, *L*. *stewartianum*, *L*. *matangense*, *L*. *lophophorum,* and *L*. *nanum* in the phylogenetic trees of ITS did not form a clade, which may be due to the insufficient number of ITS samples and the limited information contained in the ITS sequence. A close relationship has been shown among the six species (*L*. *lankongense*, *L*. *duchartrei*, *L*. *stewartianum*, *L*. *matangense*, *L*. *lophophorum*, and *L*. *nanum*) reflected in the plastid genome datum.

The revolute tepals of *L. lankongense*, *L. duchartrei*, *L. stewartianum*, *L. matangense* are different from those of the campanulate flowers of *L. lophophorum* and *L. nanum* (Fig. 1). However, the morphological characteristics of *Lilium* do not reflect the actual interspecies relationships. Convergent evolution, divergent evolution, retention and loss of ancestral characteristics, hybridization, and non-one-to-one correspondence between genes and morphological traits may affect plant’s morphology. The results of these five factors may be different morphologies of closely related species and similar morphologies of distantly related species, which confuses the judgment of interspecies relationships (Givnish et al., 2020). The habitats of *L. lophophorum* and *L. nanum* are mainly alpine grasslands and the adjacent niche at higher altitudes. These habitats often experience torrential downpours and have strong ultraviolet rays. The nodding and campanulate flowers can protect the reproductive structures against heavy rainfall and ultraviolet rays (Gao, Harris & He, 2015). The other four species found in the regions of lower altitudes have flowers with a larger opening, due to the revolute tepals, which may be more beneficial to pollination. The common floral characteristics of these six *Lilium* species are the inner tepals with projections on both surfaces of the nectaries, however the shape of the projections is not exactly the same. The projections of *L. lankongense* and *L. duchartrei* are papillose, *L. stewartianum* has cristate projections, and *L. matangense*, *L. lophophorum,* and *L. nanum* have fimbriate projections (Fig. S2). *L. lankongense* and *L. duchartrei* have habitats in lower elevations than the other four species and their inner and outer tepals have papillose projections. The other four species have habitats at higher elevations and only have projections in the inner tepals. *L. stewartianum* grows in open and rocky places on limestone mountains or along valleys and forest margins. Its habitat is distinct from those of the other three species, and the cristate projections are also different. *L. matangense* grows on grassy slopes, *L. lophophorum* and *L. nanum* grow mainly on bushy slopes and alpine grasslands. These three species have similar habitats and their projections are fimbriate. We speculate that the adaptation to different habitats may cause the dissimilarities of floral morphology among *L*. *lankongense*, *L*. *duchartrei*, *L*. *stewartianum*, *L*. *matangense*, *L*. *lophophorum,* and *L*. *nanum.*

Although the flower morphology of *L*. *lophophorum* and *L*. *nanum* was different from the other four species, they shared similar karyotypes that were measured using MAML (Altinordu et al., 2016). Gao, Zhou & He (2011) found that *L*. *lophophorum*, *L*. *nanum*, and *L*. *matangense* held a 3A type and relative asymmetry index and there were multiple, approximative parameters of the karyotype between *L*. *stewartianum* and *L*. *lophophorum* (Wan et al., 2011). The phylogenetic trees in this study demonstrated that the four species were more closely related (Figs. 6, 7, and Fig. S1). *L*. *matangense*, *L*. *lophophorum*, and *L*. *nanum* had karyotypes that resembled a clade formed by *L*. *duchartrei* and *L*. *lankongense* (Gao et al., 2012a). Therefore, it is reasonable to conclude that these six species constituted a clade. *L*. *duchartrei* and *L*. *lankongense*, which are closely related (Jefferson-Brown & Howland, 1995; MacRae, 1998), were thought to be one species (Wang & Tang, 1980), but were later separated due to the different colors of the tepal (*L. lankongense*: pink tepal*, L. duchartrei*: white tepal), the obvious geography variations (within the Daxue Mountains), and the significant genetic differentiation of the 22 populations (Gao et al., 2013; Liang & Tamura, 2000; Shen, Zhou & He, 2014). There are obvious micromolecular differences in the leaf dermis such as the inner margin and the waxy ornamentation of the outer stomatal rim, and the characteristics of the cuticular membrane under the electron microscope (*L. lankongense*: the pattern of anticlinal wall of leaf epidermal cells are all straight and the guard cells have no T pieces; *L. duchartrei*: the pattern of anticlinal wall of leaf epidermal cells are wavy and the guard cells have T pieces at the two poles) (Zhou, 2008). Our research supports the classification of *L. lankongense* as an independent species and agrees with the classification of Flora of China (Liang & Tamura, 2000). The two species have been clustered in the same clade based on the internal transcribed spacer region or plastid genes (Du et al., 2014; Gao et al., 2012a; Givnish et al., 2020; Lee et al., 2011; Nishikawa et al., 2001) (Figs. 6, 7, and Fig. S1). *L*. *duchartrei* also showed a sister relationship with *L*. *lankongense* in the phylogenetic trees of ITS and plastid genomes, which is in agreement with results from previous studies (Figs. 6, 7, and Fig. S1). We reconstructed the phylogenetic relationship of *Lilium* with 33 plastid whole genomes. However, we only conducted a preliminary analysis of the relationship of some species in *Lilium* due to the limited plastid genome datum. More plastid genomes should be obtained to better clarify the classification at the section level of *Lilium* and the relationship among species.

## Conclusions

The plastid genomes of the six *Lilium* species shared a high similarity in genome organization, order, and content, however the IR and single copy boundaries were slightly inconsistent. The total number of SSRs ranged from 53 to 63. We designed a series of new primers by the conservative regions learned from all available *Lilium* plastid genomes alignments. Two *Lilium*-specific primer pairs were verified to be effective. Two intergenic spacer regions (*trnC*-*petN* and *rpl32*-*trnL*) can be well-amplified with the two new primer pairs and may be applied in future population-level studies of *Lilium*. The phylogenetic inference supported that *Lilium* is a monophyletic group. The phylogenetic tree of ITS is not completely consistent with the phylogenetic tree based on two data sets of the plastid genome (the plastid genome sequences, the shared 71 CDS sequences). The inconsistencies may be due to hybridization, paralogous homology of ITS sequences, the limited information contained in ITS sequences, and the small number of *Lilium* species involved in this study. The results of phylogenetic studies on plastid genomes have shown that the six species, namely *L*. *lankongense, L*. *duchartrei, L. stewartianum*, *L*. *matangense, L*. *lophophorum,* have close phylogenetic relationships.

##  Supplemental Information

10.7717/peerj.10964/supp-1 Supplemental Information 1 Phylogenetic tree reconstruction of 33 species using maximum likelihood (ML) and Bayesian inference (BI) method based on concatenated sequences of shared 71 protein-coding genes from different species Species of the *L. lankongense* and five related species are highlighted in red branches. The color patches highlight species in each of the traditional sections and Nomoc*haris* or *Lilium duchartreis* clade. Bootstrap support values in the ML trees and posterior probabilities in the BI trees are shown at the corresponding nodes.

10.7717/peerj.10964/supp-2 Supplemental Information 2 Habitats, inner and outer tepals, enlarged view of projections of inner tepals in *L. lankongense* and its 5 related species Scale bar indicating two mm.

10.7717/peerj.10964/supp-3 Supplemental Information 3 Sample information of six *Lilium* species

10.7717/peerj.10964/supp-4 Supplemental Information 4 Primer sequences and amplification results Primers were designed using genes alignment of all published plastid genome sequences in *Lilium*. For each primer pair, forward (F) and reverse (R) primers are indicated. Amplifications were tested on different *Lilium* species. Ok = good amplification; No = partial or no amplification.

10.7717/peerj.10964/supp-5 Supplemental Information 5 List of genes in complete plastid genomes of six Lilium species

10.7717/peerj.10964/supp-6 Supplemental Information 6 Statistics of simple sequence repeats in the six *Lilium* species Marked red repeats are common SSR to the six *Lilium* species.

10.7717/peerj.10964/supp-7 Supplemental Information 7 Information of *Lilium* species for amplification and sequence

10.7717/peerj.10964/supp-8 Supplemental Information 8 The mutation sites were displayed among different populations of three *Lilium* species, based on the amplified sequences of the two pairs of primers derived from the *trnC-petN* and *rpl32-trnL* regions

10.7717/peerj.10964/supp-9 Supplemental Information 9 The rpl32-trnL fragment Nucleotide alignment of 11 raw sequence and gene accession numbers

10.7717/peerj.10964/supp-10 Supplemental Information 10 The trnC-petN fragment Nucleotide alignment of 11 raw sequence and gene accession numbers

## Additional Information and Declarations

### DNA Deposition

The following information was supplied regarding the deposition of DNA sequences: The plastid and ITS sequences described here are available at GenBank: MK757466, MN745200, MN745202, MN745201, MK493298, MK493300, MT260888, MT260889, MT260890, MT260891, MT260892, and MT260893.

### Data Availability

The following information was supplied regarding data availability: The raw measurements are available in the Supplemental Files. All Voucher specimens are deposited in SZ (Sichuan University Herbarium). The herbarium number of *L. lankongense, L. duchartrei, L. matangense, L. stewartianum, L. lophophorum*, and *L. nanum* are respectively KS2018071601, KS20180704, LS20180622, KS2018070203, LJ2017062301, YM20140828-3.
