# Supplementary figures and images for: Comparative analysis of complete plastid genomes from Lilium lankongense Franchet and its closely related species and screening of Lilium-specific primers
Source: PeerJ. 2021 Mar 5;9:e10964. doi: 10.7717/peerj.10964 (PMC7938781; doi:10.7717/peerj.10964)

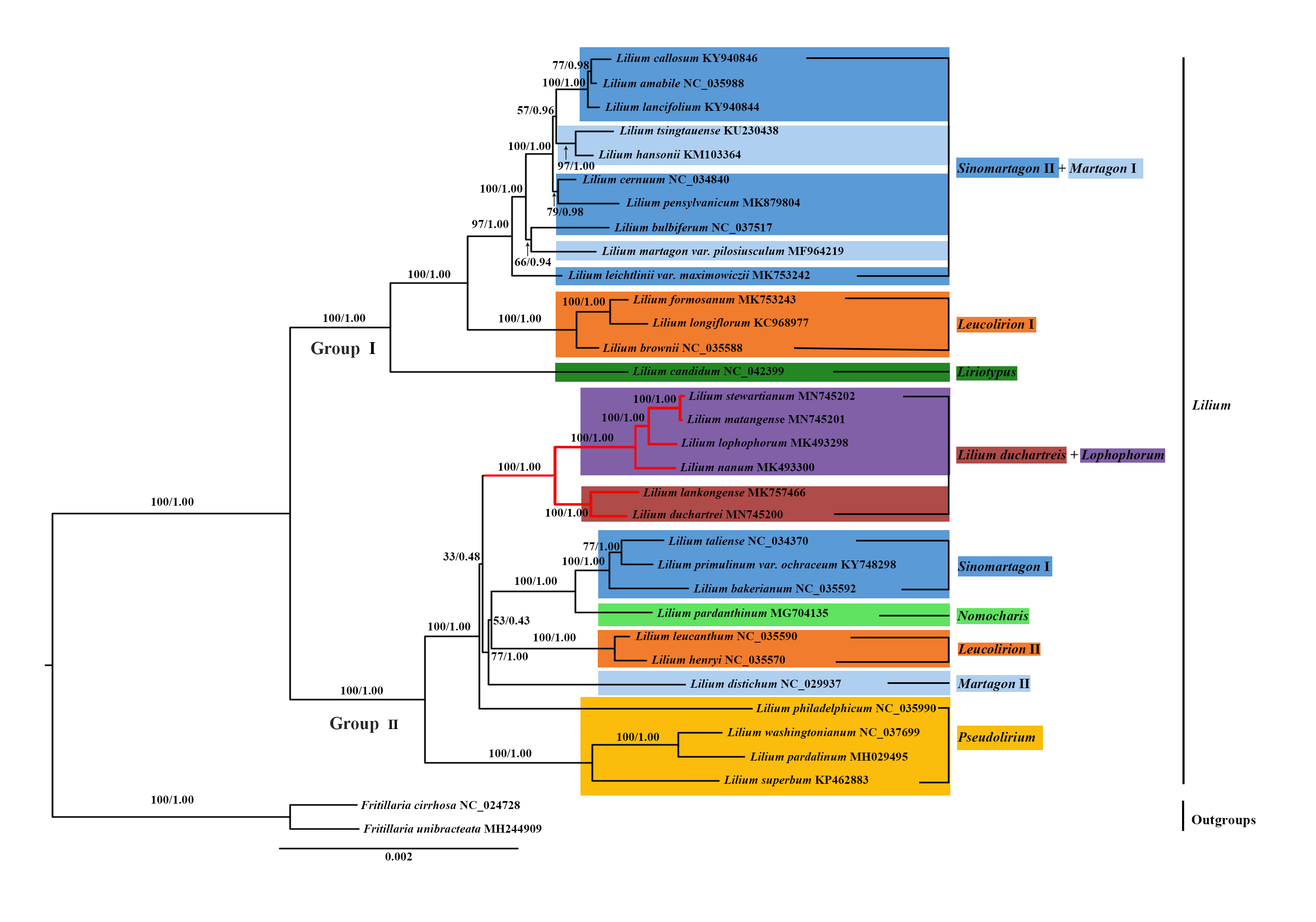

Supplement: Supplemental Information 1 — Species of the L. lankongense and five related species are highlighted in red branches. The color patches highlight species in each of the traditional sections and Nomocharis or Lilium duchartreis clade. Bootstrap support values in the ML trees and posterior probabilities in the BI trees are shown at the corresponding nodes. [file peerj-09-10964-s001.png]

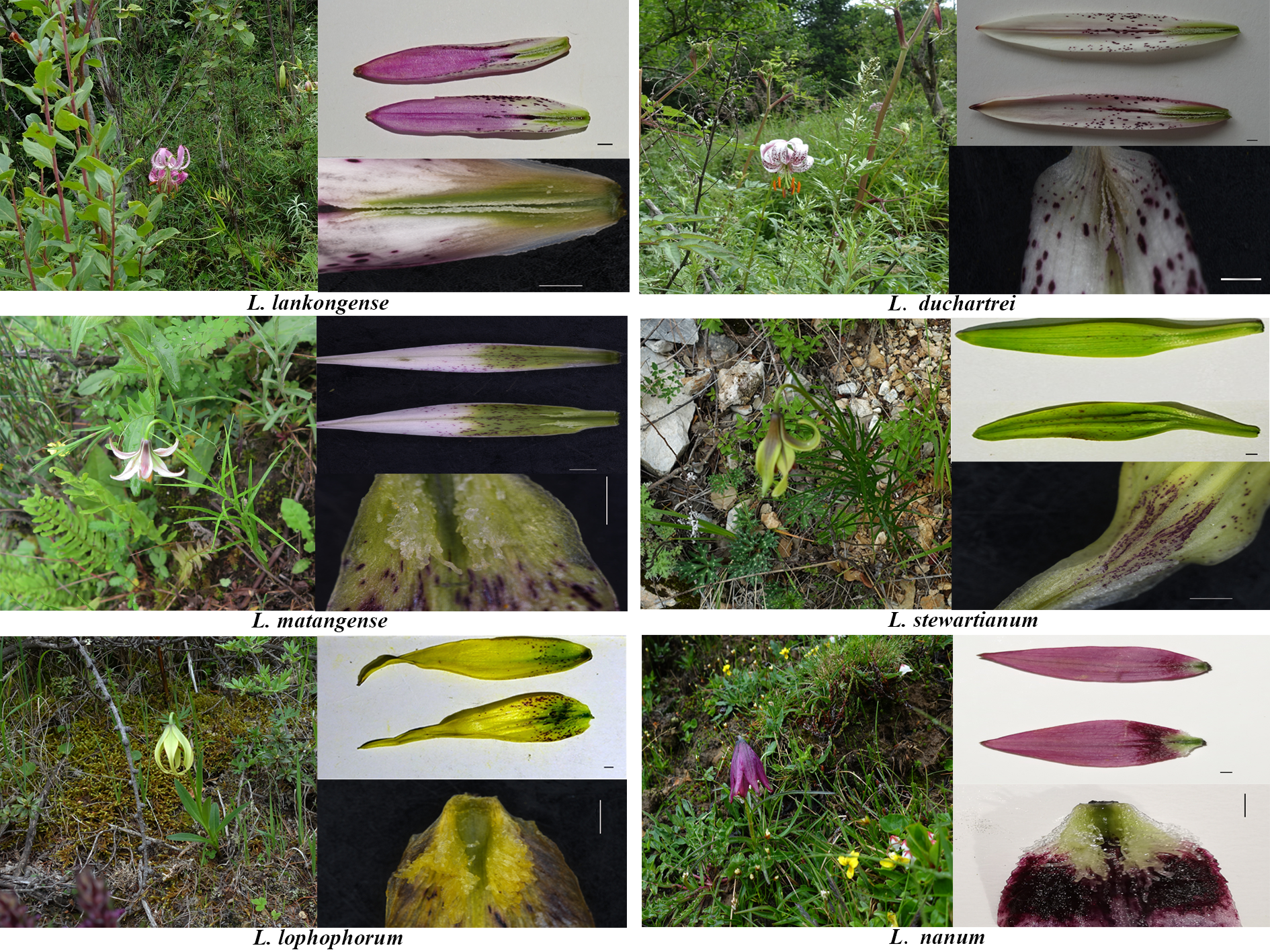

Supplement: Supplemental Information 2 — Scale bar indicating two mm. [file peerj-09-10964-s002.png]
