# Supplementary material for: Comparative analysis of complete plastid genomes from Lilium lankongense Franchet and its closely related species and screening of Lilium-specific primers
Source: PeerJ. 2021 Mar 5;9:e10964. doi: 10.7717/peerj.10964 (PMC7938781; doi:10.7717/peerj.10964)
Supplement: Supplemental Information 3 [file peerj-09-10964-s003.docx]

| **Species** | **Sampling locality** | **Latitude**  **(N)** | **Longitude**  **(E)** | **Voucher**  **(SZ)** |
| --- | --- | --- | --- | --- |
| *L. lankongense* | Lijiang, Yunnan | 27°12′07″ | 99°26′42.1″ | KS2018071601 |
| *L. duchartrei* | Kangding, Sichuan | 30°07′38.40″ | 101°55′36.6″ | KS20180704 |
| *L.* *stewartianum* | Xiangcheng, Sichuan | 28°57′58.14″ | 99°45′29.09″ | SDM2018070203 |
| *L. matangense* | Markang, Sichuan | 31°54′15.53″ | 102°38′57.09″ | LS20180622 |
| *L. lophophorum* | Jinchuan, Sichuan | 31°28′40.87″ | 102°03′40.96″ | LJ2017062301 |
| *L. nanum* | Nyingchi , Tibet Autonomous Region | 29°37′27.19″ | 94°39′2.22″ | YM20140828-3 |
