# Supplementary material for: Comparative analysis of complete plastid genomes from Lilium lankongense Franchet and its closely related species and screening of Lilium-specific primers
Source: PeerJ. 2021 Mar 5;9:e10964. doi: 10.7717/peerj.10964 (PMC7938781; doi:10.7717/peerj.10964)
Supplement: Supplemental Information 5 [file peerj-09-10964-s005.docx]

| **Category for genes** | **Group of genes** | **Name of genes** |
| --- | --- | --- |
| **Self-replication** | transfer RNAs | *trnA-UGC**, *trnC-GCA*, *trnD-GUC*, *trnE-UUC*, *trnF-GAA*, *trn*f*M-CAU*, *trnG-GCC*, *trnG-UCC*, *trnH-GUG**, *trnI-CAU**, *trnI-GAU**, *trnK-UUU*, *trnL-CAA**, *trnL-UAA*, *trnL-UAG*, *trnM-CAU*, *trnN-GUU**, *trnP-UGG*, *trnQ-UUG*, *trnR-ACG**, *trnR-UCU*, *trnS-GCU*, *trnS-GGA*, *trnS-UGA*, *trnT-GGU*, *trnT-UGU*, *trnV-GAC**, *trnV-UAC*, *trnW-CCA*, *trnY-GUA* |
|  | ribosomal RNAs | *rrn4.5**, *rrn5**, *rrn16**, *rrn23** |
|  | RNA polymerase | *rpoA*, *rpoB*, *rpoC1*, *rpoC2* |
|  | Small subunit of ribosomal proteins (SSU) | *rps2*,*rps3*, *rps4*, *rps7**, *rps8*, *rps11*, *rps12**, *rps14*, *rps15*, *rps16*, *rps18*, *rps19** |
|  | Large subunit of ribosomal proteins (LSU) | *rpl2**, *rpl14*, *rpl16*, *rpl20*, *rpl22*, *rpl23**, *rpl32*, *rpl33*, *rpl36* |
| **Genes for photosynthesis** | Subunits of NADH-dehydrogenase | *ndhA*, *ndhB**, *ndhC*, *ndhD*, *ndhE*, *ndhF*, *ndhG*, *ndhH*, *ndhI*, *ndhJ*, *ndhK* |
|  | Subunits of photosystem I | *psaA*, *psaB*, *psaC*, *psaI*, *psaJ* |
|  | Subunits of photosystem II | *psbA*, *psbB*, *psbC*, *psbD*, *psbE*, *psbF*, *psbH*, *psbI*, *psbJ*, *psbK*, *psbL*, *psbM*, *psbN*, *psbT*, *psbZ* |
|  | Subunits of cytochrome b/f complex | *petA*, *petB*, *petD*, *petG*, *petL*, *petN* |
|  | Subunits of ATP synthase | *atpA*, *atpB*, *atpE*, *atpF*, *atpH*, *atpI* |
|  | Large subunit of rubisco | *rbcL* |
| **Other genes** | Protease | *clpP* |
|  | Maturase | *matK* |
|  | Subunit of Acetyl-CoA-carboxylase | *accD* |
|  | Envelope membrane protein | *cemA* |
|  | C-type cytochrome synthesis gene | *ccsA* |
| **Genes of unknown function** | hypothetical chloroplast reading frames (ycf) | *ycf1**, *ycf2**, *ycf3*, *ycf4* |
